# Supplementary material for: Natural Variation of StNADC Regulates Plant Senescence in Tetraploid Potatoes (Solanum tuberosum L.)
Source: Int J Mol Sci. 2025 May 5;26(9):4389. doi: 10.3390/ijms26094389 (PMC12072370; doi:10.3390/ijms26094389)
Supplement: Supplementary file 1 [file ijms-26-04389-s001.zip › ijms-3567604-supplementary.pdf]

Supplementary Information

StNADC Plays an Indispensable Role for NAD Synthesis and Regulates Senescence in Tetraploid Potatoes (Solanum tuberosum L.)

Jaojiao Zhang[1,2], Jianfei Xu[1], Chunsong Bian[1], Shaoguang Duan[1], Jun Hu[1], Junhong Qin[1], Huan Wu[1], Ming He[1], Yinqiao Jian[1], Yanfeng Duan[1], Jiangang Liu[1], Wanxing Wang[1], Guangcun Li[1]*, Liping Jin[1]*

[1.] Institute of Vegetables and Flowers, Chinese Academy of Agricultural Sciences/ State Key Laboratory of Vegetable Biobreeding /Key Laboratory of Biology and Genetic Improvement of Tuber and Root Crop of Ministry of Agriculture and Rural Affairs, Beijing 100081, China.

[2.] ZJU-Hangzhou Global Scientific and Technological Innovation Center. Innovation Research Institute, Hangzhou 310000, China.

*Corresponding author. Prof. Guangcunli (E-mail: liguangcun@caas.cn); Prof. Liping Jin (Jinliping@caas.cn)

State Key Laboratory of Vegetable Biobreeding, Institute of Vegetables and Flowers, Chinese Academy of Agricultural Sciences/ Beijing 100081, China.

Figure S1. Expression profiles of *StNADC*. Expression of *StNADC* in various organs **(A)** and different age leaves **(B)** from Z3 and Z19 plants. The relative expression levels are normalized to *ELF3e.* The data are means ± SE of three biological replicates.

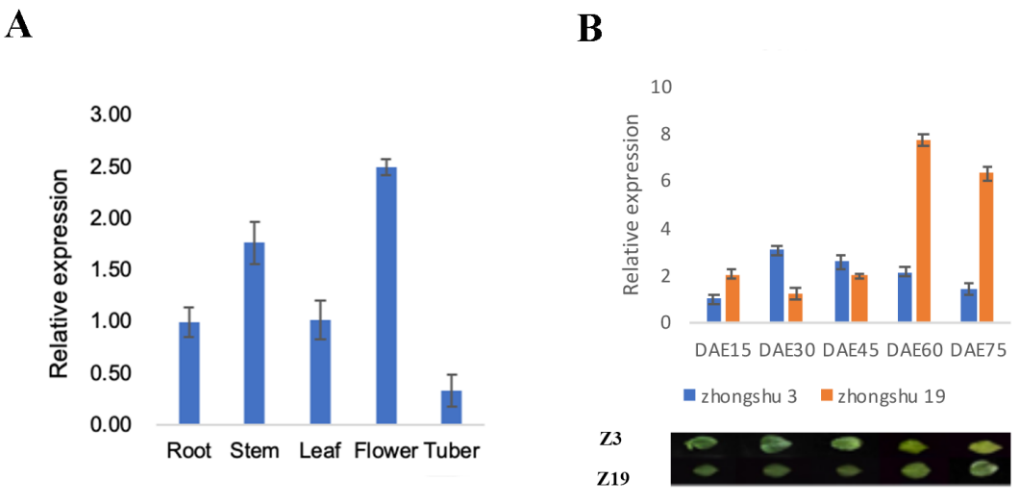

A

Differences nucleotide sequence of *St NADC* between early and late maturing parents

Haplotype	Dosage	21	28	33	40	105	297	314	324	353	409	584	674	725	809	938
H1(Z3-7)	4	A	C	T	G	A	A	G	C	G	A	T	T	A	T	G
H2(Z3-68&Z119-28)	8+7	C	T	C	A	A	G	A	C	C	A	T	A	G	T	A
H3(Z3-42&Z19-29)	2+3	A	C	C	G	A	G	G	T	G	A	T	T	A	T	A
H4(Z3-57&Z19-32)	2+2	A	C	C	G	G	G	G	T	G	G	C	T	A	C	A

B

Amino acid sequence differences between early and late maturing parents

Haplotype	Dosage	7	9	11	13	35	99	108	117	136
H1(Z3-7)	4	T	T	Y	C	T	T	F	K	H
H2(Z3-68&Z119-28)	8+7	P	I	H	Y	T	A	F	N	H
H3(Z3-42&Z19-29)	2+3	T	T	H	C	T	A	S	K	H
H4(Z3-57&Z19-32)	2+2	T	T	H	C	A	A	S	K	R

Figure S2. **Sequence analysis of *StNADC*.**

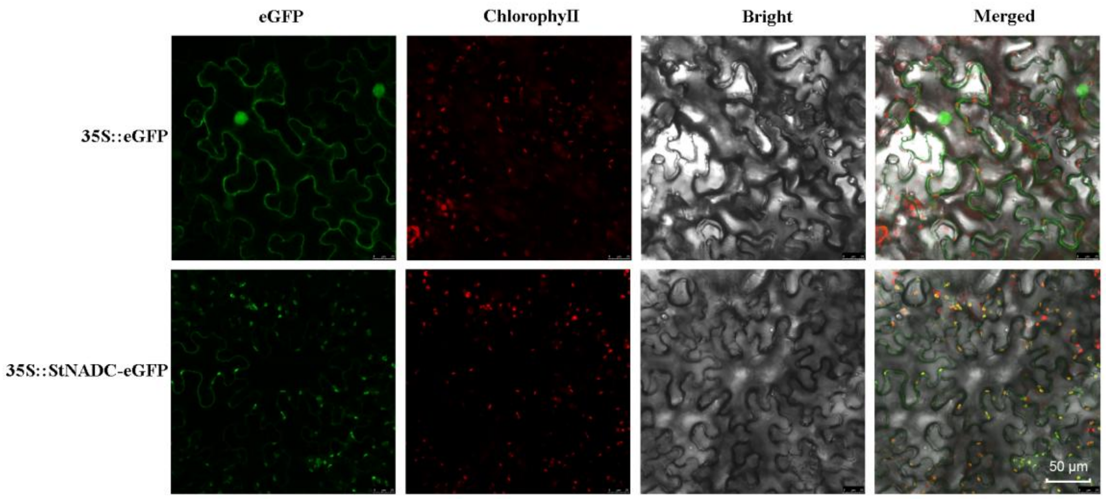

Figure S3. **Subcellular localization of *StNADC-eGFP* fusion protein located at chloroplast.**

Transient expression of 35S::eGFP and 35S::*StNADC*-eGFP fusion *N. benthamiana* leaves were photographed under a confocal microscope at 488 nm. eGFP, eGFP fluorescence, Chlorophyll,

Chlorophyll autofluorescence, Bright, Bright field image, Merge, Superposition of eGFP, Chlorophyll, and bright field. Scale bar, 10μm.

Figure S4. **Summary of Metabolome and Transcriptome Data of the *cr2-11* and wild type.**

(A) Two stages of WT and *cr2-11* simples for metabolome and transcriptome analysis. (B) Overview classification of 318 annotated metabolites. (C) Categories of DEMs between WT and *cr2-11* mutant. (D) Heat maps of DEGs between WT and *cr2-11* mutant. (E) the number of DEGs between cr2-11 and wild type in Day15 and Day30. d, the number of DEGs between cr2-11 and wild type in Day 15 and Day 30.

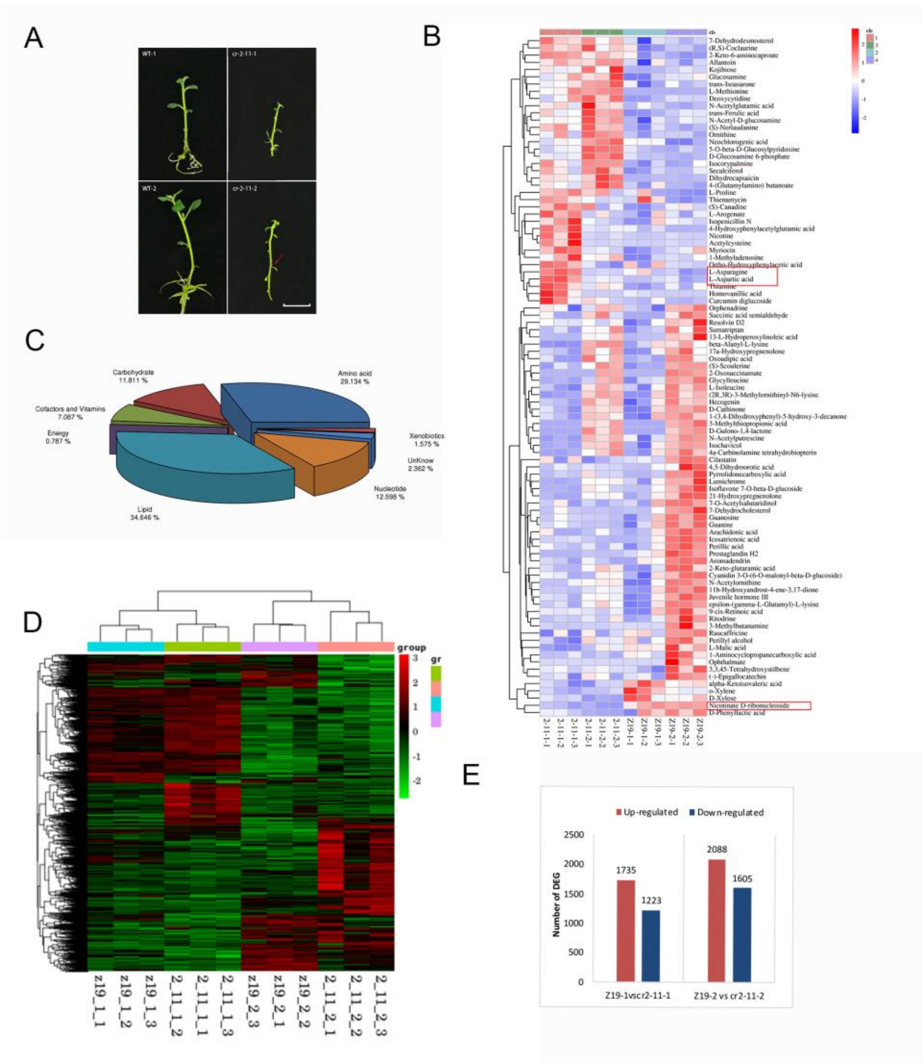

Figure S5. qPCR validations of the transcriptome.

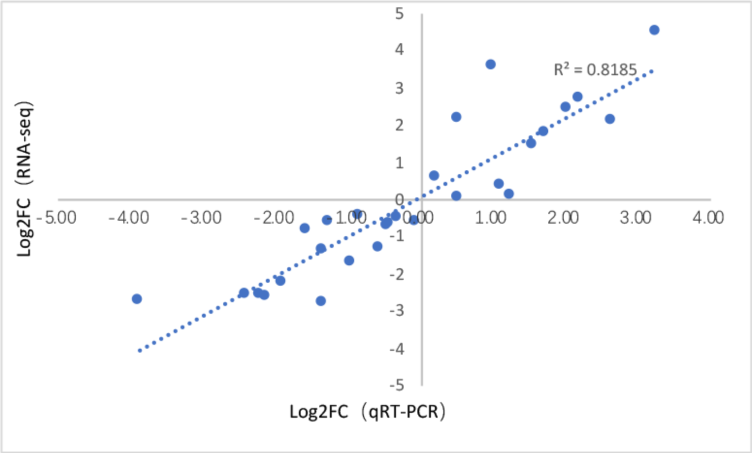

Figure S6. KEGG analysis of ME green and ME blue module. (A) Down-regulate genes of *cr2-11* compared to WT in the green module. (B) KEGG analysis of genes in the green module. (C) High-regulate genes in Blue module. (D)KEGG analysis of genes in blue module.

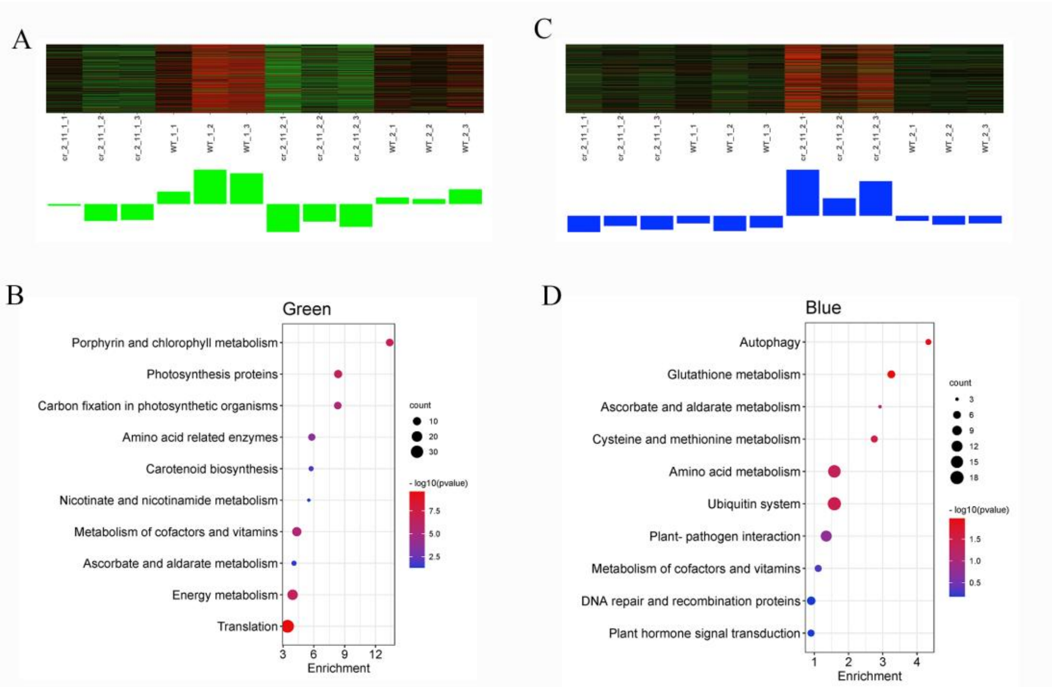

Table S1. Concordance of KASP markers with senescence in segregate population and natural population.

Material	Senescence type	Marker Name									
		c5_4031675		c5_4041510		c5_4058695		c5_4114205		c5_4116968	
Segregate	Early	79/91	0.87	48/53	0.91	78/90	0.87	46/50	0.92	48/52	0.92
Population	Late	82/101	0.81	51/62	0.82	74/95	0.78	51/58	0.88	50/60	0.83
Nature population	Early	27/35	0.77	37/45	0.82	27/39	0.69	27/42	0.64	29/39	0.74
	Late	21/36	0.58	22/44	0.5	23/51	0.45	30/44	0.68	19/43	0.44

Table S2. Genotyping of recombinant progeny.

标记	中薯3号	中薯19号	早熟						晚熟						
			67	147	49	55	85	11	142	172	179	213	31	100	6
c5_4029280	1	0	1	1	1	1	1	1	0	0	0	0	0	0	1
c5_4041510	1	0	1	1	1	1	1	1	0	0	0	0	0	0	1
c5_4114205	1	0	1	1	1	1	1	1	0	0	0	0	0	0	0
c5_4116968	1	0	1	1	1	1	1	1	0	0	0	0	0	0	0
CAPS5-24	1	0	1	1	1	1	0	0	0	0	0	0	1	1	0
SCAR5-3-2	1	0	1	1	1	1	0	0	0	0	0	0	1	1	0
SCAR5-5	1	0	1	1	1	1	0	0	0	0	0	0	1	1	0
SCAR5-8	1	0	1	1	1	1	0	0	0	0	0	0	1	1	0

Table S3. Information of candidate genes associated with potato senescence.

Gene ID (PESC V4.03)	Gene ID (DM6.1)	Gene function
PGSC0003DMG400030550	Soltu.DM.05G004680	StEIF6 Myb-contain
PGSC0003DMG400030549	Soltu.DM.05G004690	SERGT1Conserved gene of unknown function
PGSC0003DMG400030548	Soltu.DM.05G004700	Myb-like transcription factor 6
PGSC0003DMG400030547	Soltu.DM.05G004720	E3 ubiquitin ligase PUB14
PGSC0003DMG400030504	Soltu.DM.05G004730	Conserved gene of unknown function
PGSC0003DMG400030503	\	Conserved gene of unknown function
PGSC0003DMG400030546	\	Conserved gene of unknown function
PGSC0003DMG400030502	Soltu.DM.05G004760	Fyve finger-containing phosphoinositide kinase, fyv1
PGSC0003DMG400030582	\	Gene of unknown function
PGSC0003DMG400030544	Soltu.DM.05G004770	WD-repeat protein
PGSC0003DMG400030501	Soltu.DM.05G004780	Quinolinate phosphoribosyl transferase

Table S4. Summary data for Transcriptome.

Sample	Clean Data （bp）	Reads No.	N （%）	Q20 （%）	Q30 （%）	Clean Reads No.	Clean Reads%	Uniquely Mapped
2_11_1_1	6790320000	49216088	0.000444	97.99	94.24	45268800	91.97	38776058 （96.11%）
2_11_1_2	6273855000	45549506	0.000453	98.1	94.48	41825700	91.82	35634103 （96.27%）
2_11_1_3	6546134700	47620976	0.00045	98.18	94.59	43640898	91.64	37312044 （96.33%）
z19_1_1	7176448500	51807370	0.000446	98	94.2	47842990	92.34	40885742 （96.09%）
z19_1_2	6461559600	46821484	0.000449	98.17	94.65	43077064	92	36852177 （95.69%）
z19_1_3	6198002700	44923842	0.000452	98.1	94.5	41320018	91.97	35290140 （95.65%）
2_11_2_1	6921742500	50024116	0.000447	98.11	94.52	46144950	92.24	38990867 （95.97%）
2_11_2_2	6810164400	49112532	0.000446	98.11	94.51	45401096	92.44	38045955 （96.35%）
2_11_2_3	5998301400	43269100	0.000451	98.04	94.37	39988676	92.41	33363448 （96.00%）
z19_2_1	6363598500	45972514	0.000449	98.15	94.64	42423990	92.28	36382605 （96.16%）
z19_2_2	6280779900	45239952	0.000451	98.14	94.65	41871866	92.55	35852738 （96.04%）
z19_2_3	6583908300	47450042	0.000451	98.05	94.34	43892722	92.5	37552645 （95.94%）

Table S5. Sequence information for KASP markers.

SNP_ID	PrimerName	Sequence
c5_4058695	c5_4058695-X	GAAGGTGACCAAGTTCATGCTCAGAGCATAATTTGGGGTAAGCGTA
	c5_4058695-Y	GAAGGTCGGAGTCAACGGATTGAGCATAATTTGGGGTAAGCGTG
	c5_4058695_C	CATGCCCAAAAACTCAGCAACAACTC
c5_4031675	c5_4031675-X	GAAGGTGACCAAGTTCATGCTAACTACTGGGAAGCCATAGGTG
	c5_4031675-Y	GAAGGTCGGAGTCAACGGATTCAAACTACTGGGAAGCCATAGGTT
	c5_4031675_C	TTGGATCTATTCCTGCCTTGTGAGC
c5_4041510	c5_4041510-X	GAAGGTGACCAAGTTCATGCTTTCAGGCCTTGAGGAATGATGAAATA
	c5_4041510-Y	GAAGGTCGGAGTCAACGGATTCAGGCCTTGAGGAATGATGAAATG
	c5_4041510_C	TTGGGAGGATTCCTGAGAACTATTTTTG
c5_4116968	c5_4116968-X	GAAGGTGACCAAGTTCATGCTGGGATTGTAGCAGGAATTGCACTTA
	c5_4116968-Y	GAAGGTCGGAGTCAACGGATTGGATTGTAGCAGGAATTGCACTTG
	c5_4116968_C	CCGAATTGAAAAAATCAATACCTTTAGTGAAG
c5_4114205	c5_4114205-X	GAAGGTGACCAAGTTCATGCTCGAAATGTAGGTAACACCAGTTTGC
	c5_4114205-Y	GAAGGTCGGAGTCAACGGATTTCGAAATGTAGGTAACACCAGTTTGT
	c5_4114205_C	GTTACCCTTGAAACAGTGCACGCAATT

Table S6. Primer sequences used in real-time RT-PCR expression of senescence genes.

Primer name	Sequence	Product length
04G003210F	ACTGTTGGCTCTGTCCTTTC	178
04G003210R	CACCTTTGCTCTCAACATGAAC	
03G036530F	TGCTGGGAAAGCTGATCTGG	127
03G036530R	AGTGACTGCCACAAATCCGT	
06G015450F	GCATTCCTTCTGTCGCTGTA	185
06G015450R	GGCAACTTCTCGTGGTACAT	
06G015470F	GACGAAGCAAGGCAAGACTA	133
06G015470R	GGTGCTCTCTTCCACAGATG	
05G004780F	CAAATGGGGGTTGAGGTTGA	140
05G004780R	ACCTCAACATCCCCGTTAGA	
11G007490F	ATGGGTTATCGGCTTCATCATT	127
11G007490R	GCTCCATTTGCCCCTGATTT	
11G007470F	TCACAAAGGGGATTTTGCATC	156
11G007470R	ATAGGGCAAGTGGGGTGTAA	
11G007510F	ACGGCTTAGACTATGAAACAAGA	150
11G007510R	AACAGAGATGAGTCGCAAGC	
StE3-10G029510F	ATATGGAACGGAGGAGTCCAA	155
StE3-10G029510R	ATGCAAGAAATGGCCTCTGA	
P5G-05G024030F	TTGTACACTACGAGAGCCCA	171
P5G-05G024030R	CAGCAACAGGCAAACCTAGA	
sirt2-04G005960F	TGTTGTATTGACGGGAGCTG	134
sirt2-04G005960R	GCCTTCGAGCCTTGATTGAT	
CRY-04G029750F	GTCACCTGGCTGGAGTAATG	147
CRY-04G029750R	CGAACACTGACTTCCCCAAA	
FKF1-01G000490F	AAAGAAACTTGCCAGCCTCA	152
FKF1-01G000490R	GCAACATCCCTTGGAGTCAA	
GI-12G007510F	CTTCCTCCACAAGATGCAGAG	185
GI-12G007510R	TGCTCCGGGTGATGAAGAAC	
COL9-12G023800F	TGCGGTGGACTTGAGTATTG	133

COL9-12G023800R	GCTGTAGTCGGCACCTTTTA	
COL-10G027530F	TTCCTTGAAAATGCTCGCCT	166
COL-10G027530R	CAGTGTCGATCTTGGCATCA	
SERGT-05G004690F	TGGCTATACTCCACGAGAGG	197
SERGT-05G004690F	GTGCCCTCCTTTTGCTATGA	
NADkinase-06G016960F	TTGGGAGGAGATGGTGTGAT	200
NADkinase-06G016960R	CATCGGAGACGCATCCTTAG	
ElF3e -F	GGAGCACAGGAGAAGATGAAGGAG	
ElF3e-R	CGTTGGTGAATGCGGCAGTAGG	

72

Table S7. Primers used for gene clone and gene editing.

primer name	Sequence	Tm℃
pBI121GFP-XbaI-*StNADC*-F	GGAGAGAACACGGGGGACTCTAGAATGTTCAGGGTTCTTCCCTTC	58
pBI121GFP--XmaI-*StNADC*-R	GACTGACCACCCGGGGATCCTGCTCGTTTTGTACGTCGTCGTCC	
pBI121GFP-M73F	CGACAGTGGTCCCAAAGAT	58
pBI121GFP-212R2	GTGGTGCAGATGAACTTCAGG	
1300-StE3-R2	CGATACACCAAATCGACTCTAGAATGGCCGGCGGAGAAG	58
1300-StE3-R2	TGCTCACCATGGTACCATTGCGCGTGCTATGTTG	
1300-*StNADC*-F2	CGATACACCAAATCGACTCTAGAATGTTCAGGGTTCTTCCCTTC	58
1300-*StNADC*-R2	TGCTCACCATGGTACCTGCTCGTTTTGTACGTCGTC	
P402-*StNADC*-F2	ATTGCATAAGTGGGGTGTGCTGG	58
P402-*StNADC*-R2	AAACCCAGCACACCCCACTTATG	
yz402*NADC*-F2	AATGGCCACCAAGAATGCAG	58
yz402*NADC*-R2	GTCAACAGGAATTGTCGCCT	
U6-26F	TGTCCCAGGATTAGAATGATTAGGC	58
